# CPEB2 inhibit cell proliferation through upregulating p21 mRNA stability in glioma

**DOI:** 10.1038/s41598-023-50848-0

**Published:** 2023-12-29

**Authors:** Guang Zhao, Zhongjun Zhao, Mingyi Xia, Lishun Xiao, Bao Zhu, Hui Wang, Xiang Li, Jiehui Di

**Affiliations:** 1 Cancer Institute, Xuzhou Medical University, 209 Tongshan Road, Xuzhou, 221004 Jiangsu China; 2 Center of Clinical Oncology, The Affiliated Hospital of Xuzhou Medical University, 99 West Huaihai Road, Xuzhou, 221002 Jiangsu China; 3 Jiangsu Center for the Collaboration and Innovation of Cancer Biotherapy, Xuzhou Medical University, 209 Tongshan Road, Xuzhou, 221004 Jiangsu China; 4 Department of Neurosurgery, The Affiliated Hospital of Xuzhou Medical University, 99 West Huaihai Road, Xuzhou, 221000 Jiangsu China; 5 Department of Emergency Medicine, The First People’s Hospital of Kunshan, Kunshan, 215300 Jiangsu China; 6 Department of Biostatistics, School of Public Health, Xuzhou Medical University, Xuzhou, China

**Keywords:** Cancer, Neuroscience, Oncology

## Abstract

Glioma is the most common primary malignant brain tumor in adults and remains an incurable disease at present. Thus, there is an urgent need for progress in finding novel molecular mechanisms that control the progression of glioma which could be used as therapeutic targets for glioma patients. The RNA binding protein cytoplasmic polyadenylate element-binding protein 2 (CPEB2) is involved in the pathogenesis of several tumors. However, the role of CPEB2 in glioma progression is unknown. In this study, the functional characterization of the role and molecular mechanism of CPEB2 in glioma were examined using a series of biological and cellular approaches in vitro and in vivo. Our work shows CPEB2 is significantly downregulated in various glioma patient cohorts. Functional characterization of CPEB2 by overexpression and knockdown revealed that it inhibits glioma cell proliferation and promotes apoptosis. CPEB2 exerts an anti-tumor effect by increasing p21 mRNA stability and inducing G1 cell cycle arrest in glioma. Overall, this work stands as the first report of CPEB2 downregulation and involvement in glioma pathogenesis, and identifies CPEB2 as an important tumor suppressor gene through targeting p21 in glioma, which revealed that CPEB2 may become a promising predictive biomarker for prognosis in glioma patients.

## Introduction

Glioma, as the most common primary malignancy in the central nervous system (CNS), originates from glial cells of the brain and is characterized by strong genetic heterogeneity, chemotherapy resistance and high mortality^1,2^. Despite surgery, radiotherapy, and chemotherapy have been improved in glioma clinical treatment, the median survival and prognosis remains poor due to therapeutic resistance^3–5^. Therefore, it is necessary to dissect the inner molecular changes that underlie glioma progression and identify novel potential biomarkers for the treatment of glioma.

Reprogramming of gene expression is a key step during many processes of tumor development^6,7^. Cytoplasmic Polyadenylation Element Binding Protein 2 (CPEB2) is a highly conserved, sequence-specific RNA binding protein that belongs to the CPEB family^8,9^. CPEB family proteins (CPEBs) control the translation of various genes and have been reported in association with biological malignancy in cancers^8,9^. CPEBs are composed of four members CPEB1-4 and all CPEBs harbor a conserved C-terminal region composed of two RNA recognition motifs (RRMs) and two zinc finger (ZF) domain^10,11^. Of which, the RRMs of CPEB2–4 share 97% sequence identity between them^12^. CPEB1-4 are differentially expressed in somatic tissues but they appear to regulate overlapping populations of mRNAs^13–15^.

CPEB1 and CPEB3 were shown to be a tumor-suppressor^16–19^, while CPEB4 seems to play paradoxical roles in different cancers^20–22^. The role of CPEB2 in cancer appear to be more complex and remains paradoxical. A tumor-suppressor role of CPEB2 was suggested by binding to the mesenchymal transcription factor TWIST1 and suppressing its translation^23^, and was further suggested by its down-regulation by microRNA-885-5p, which is a mediator of EMT, tumorigenesis and metastasis^24^. The roles of CPEB2 in breast cancer depend on the expression of different CPEB2 isoforms. Park et al. demonstrated that alternative splicing of CPEB2 mRNA enhanced the metastasis of Triple-negative breast cancer (TNBC) cells, while CPEB2A (−exon4) and CPEB2B (+exon4) have the opposite function in breast cancer metastasis^25,26^.

Several reports have revealed that the upregulation of CPEB4 is clearly related with glioma cell proliferation, metastasis, and TMZ resistance^27–29^. CPEB3 protein content was increasing with WHO grade while expression of active phospho-CPEB3 protein was decreasing with tumor grade in human gliomas. Furthermore, reports on the expression and the function of CPEB1 in glioma are inconsistent^17,30^. Different member of CPEBs seems to play paradoxical roles in glioma, and so far, little was known about the correlation between CPEB2 expression and glioma pathogenesis, and the prognostic significance of CPEB2 in glioma remains unclear.

In the current study, we first analyzed the expression levels of CPEB2 in glioma patient cohorts and identified CPEB2 to be significantly downregulated. We further studied its role in glioma cell proliferation by CPEB2 overexpression and knockdown. We conclude that CPEB2 is a novel tumor suppressor gene, inhibiting cancer hallmarks of proliferation while promoting apoptosis in glioma cells. Mechanistically, CPEB2 upregulates p21 expression by increasing its mRNA stability, thus further inducing cell cycle arrest. Taken together, our findings indicate that CPEB2 could be served as a new anticancer therapeutic target in glioma treatment.

## Results

### CPEB2 is downregulated in glioma tissues

**Figure 1:**
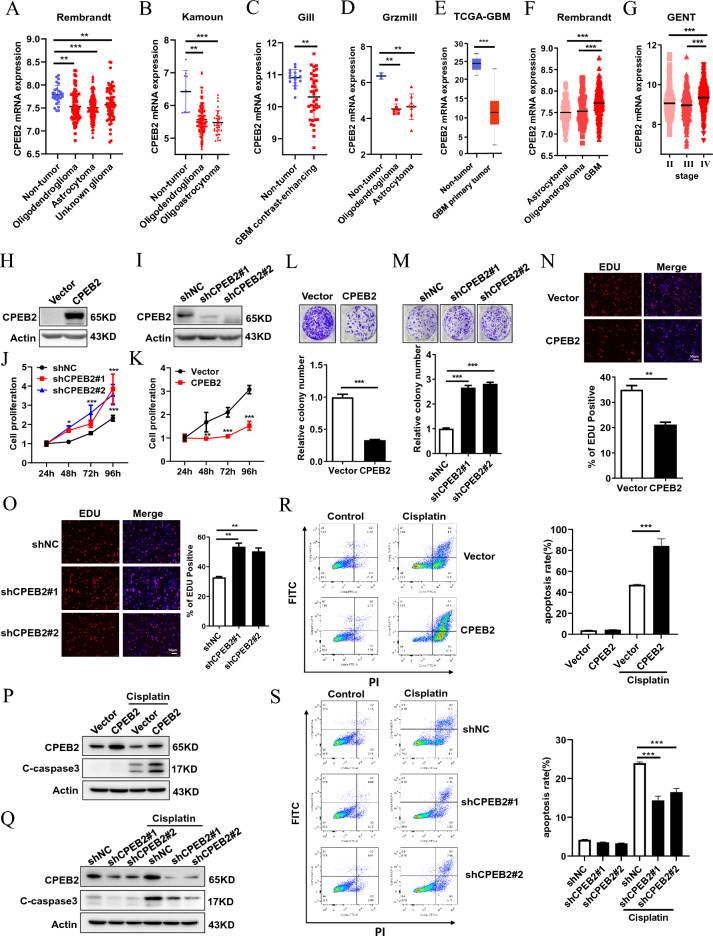
CPEB2 is downregulated in glioma tissues and inhibits cell proliferation and promotes apoptosis in U87 cells. GlioVis (a web application for exploring brain tumors expression datasets) and GENT2 database was used for expression analysis of CPEB2 in glioma patients. (**A**–**D**) Analysis of CPEB2 expression in glioma in Rembrandt dataset (Non-tumor n = 28, Oligodendroglioma n = 67, Astrocytoma n = 147, Unknown glioma n = 65) (**A**), Kamoun dataset (Non-tumor n = 9, Oligodendroglioma n = 114, Oligoastrocytoma n = 32) (**B**), Gill dataset (Non-tumor n = 17, GBM contrast-enhancing n = 38) (**C**), and Grzmill dataset (Non-tumor n = 2, Oligodendroglioma n = 6, Astrocytoma n = 8) (**D**). (**E**) Graph showing the change in expression of CPEB2 in a cohort of 156 glioblastoma patients versus non-tumor control (n = 5) in TCGA-GBM database. (**F**) Expression of CPEB2 across different subtypes of gliomas. Graph from GlioVis showing lower expression of CPEB2 in astrocytoma and oligodendroglioma as compared to glioblastoma in Rembrandt dataset (Oligodendroglioma n = 67, Astrocytoma n = 147, GBM n = 218). (**G**) Graph from GENT database showing lower expression of CPEB2 in grade II (n = 239) and grade III (n = 239) tumors as compared to grade IV (n = 451). (**H**,**I**) The efficiency of CPEB2 overexpression and knockdown in U87 cells. **J** Cell growth of U87 cells with CPEB2 overexpression or not was detected by CCK-8 assays after 1, 2, 3 and 4 days (n = 5). (**K**) Cell growth of U87 cells with CPEB2 knockdown or not was detected by CCK-8 assays after 1, 2, 3 and 4 days (n = 5). (**L**) Colony formation of U87 cells with CPEB2 overexpression or not. (**M**) Colony formation of U87 cells with CPEB2 knockdown or not. (**N**, **O**) Indicated U87 cells were stained with EDU and DAPI. The red color indicates EDU-positive nuclei. The statistical analysis of EDU staining was performed by Image-Pro Plus 6.0 software (n = 3). Column graph was mean ± SEM of three independent experiments. (**P**,**Q**) Western blot analysis of the expression levels of cleaved caspase 3 (C-caspaase 3). (**R**,**S**) Flow cytometry detection of apoptosis with Annexin V/PI staining. U87 cells with CPEB2 overexpression or knockdown were treated with 25 μg/ml cisplatin for 24 h. Apoptotic cells were detected and quantified with flow cytometry. All values are mean ± SEM. Two way ANOVA followed by Tukey's multiple comparison test or Student t-tests were used. *P < 0.05, **P < 0.01, and ***P < 0.001. *SEM* standard error of the mean.

To investigate whether CPEB2 has de-regulation of expression in glioma patients, we first checked the alterations of CPEB2 expression in glioma patient data analyses using GlioVis which is a web application for exploring brain tumors expression datasets (http://gliovis.bioinfo.cnio.es/)^31^, and found significant down-regulation of CPEB2 in glioma patients as compared to non-tumor controls across four different patient datasets namely Rembrandt (Non-tumor n = 28, Oligodendroglioma n = 67, Astrocytoma n = 147, Unknown glioma n = 65), Kamoun (Non-tumor n = 9, Oligodendroglioma n = 114, Oligoastrocytoma n = 32), Gill (Non-tumor n = 17, GBM contrast-enhancing n = 38) and Grzmill (Non-tumor n = 2, Oligodendroglioma n = 6, Astrocytoma n = 8) (Fig. 1A–D). We also performed TCGA-GBM (GBM n = 156, nontumor n = 5) patient data analyses and found CPEB2 to be decreased in glioblastoma patients (Fig. 1E). When we compared CPEB2 expression level across different subtypes of glioma, we found that it is lower in oligodendroglioma and astrocytoma which are low-grade glial tumors as compared to glioblastoma (Fig. 1F). We further revealed that CPEB2 expression is lower in grade II and grade III tumors as compared to grade IV glioblastoma using Grade wise expression analysis in Gene Expression database of Normal and Tumor tissues 2 (GENT2) database (Fig. 1G). These observations indicate a possible involvement of CPEB2 in early events of gliomagenesis. In addition, the mutation rate of CPEB2 in glioma patients are very low in cBioPortal database (Supplementary Fig. 1). These results indicated that CPEB2 is downregulated in glioma tissues and might be functioned as a tumor suppressor gene in glioma progression.

### CPEB2 inhibits cell proliferation and promotes apoptosis in glioma

**Figure 2:**
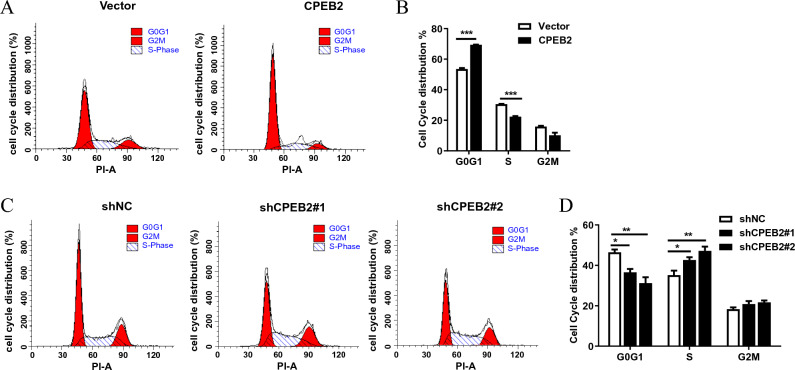
CPEB2 induces G1 cell cycle arrest. (**A**,**B**) Cell cycle distribution in U87 cells with CPEB2 stable overexpression or not. (**C**,**D**) Cell cycle distribution in U87 cells with CPEB2 stable knockdown or not. Indicated cells were starved in medium containing no serum for 48 h, and then released into complete growth medium for 24 h. After release, harvested the cells and analyzed by flow cytometry. The dataset of (**A**,**C**) is representative example of triplicate experiments. Column graph of (**B**,**D**) was mean ± SD of three independent experiments. All values are mean ± SEM. Student t-tests were used. ***P < 0.001. SEM, standard error of the mean.

To elucidate the role of CPEB2 in glioma, we checked for the effects of CPEB2 modulation on cell proliferation. For this, we performed CPEB2 stable overexpression and knockdown in U87 and U251 cell lines. The modulation of CPEB2 levels was confirmed by WB analysis (Fig. 1H,I; Supplementary Fig. 2A, D). CCK8 assay showed a decrease in proliferation upon CPEB2 overexpression while opposite results were obtained post CPEB2 knockdown (Fig. 1J,K; Supplementary Fig. 2B, E). Next, we confirmed our results by performing colony formation assay in U87 and U251 cell lines having CPEB2 modulation. Like our observation with CCK8, we found CPEB2 inhibited cell proliferation (Fig. 1L,M; Supplementary Fig. 2C, F). Similarly, the results were replicated in EdU assay (Fig. 1N,O; Supplementary Fig. 2G, H). We also checked if CPEB2 had any effects on cellular apoptosis in glioma. Western blot analysis results showed that the expression levels of cleaved caspase 3 (showed as C-caspase 3), the important indicator of apoptosis, were significantly increased by CPEB2 in U87 cells (Fig. 2P,Q). In addition, Flow cytometry analysis of cell apoptosis rates in U87 cells pretreated with Cisplatin showed that CPEB2 could promote glioma cell apoptosis (Fig. 2R,S). We also reached the same conclusion in U251 cells (Supplementary Fig. 2I, J). Collectively, these findings demonstrate that CPEB2 inhibits cell proliferation and promotes apoptosis in glioma.

### CPEB2 induces G1 cell cycle arrest in glioma

To determine whether CPEB2 can regulate cell cycle progression in glioma, we analysed cell-cycle progression using flow cytometry after using serum starvation to synchronize U87 cells. Consistent with the above results of cell proliferation assays, we found that overexpression of CPEB2 induced G1 cell cycle arrest (Fig. 2A,B), while CPEB2 knockdown promoted G1/S phase transition (Fig. 2C,D). These results suggested that CPEB2 may inhibit glioma cell growth by inhibiting G1/S transition. Then we detected the main factors involved in cell cycle regulation and found that the expression of the cyclin-dependent kinase inhibitor 1A (CDKN1A, known alternatively as p21) was up-regulated in the U87 cells with stably transfected CPEB2 (Supplementary Fig. 3A), and the data from CGGA (Chinese Glioma Genome Atlas) database showed a linear correlation between CPEB2 and p21 (Supplementary Fig. 3B).

### CPEB2 upregulates p21 expression by increasing its mRNA stability

**Figure 3:**
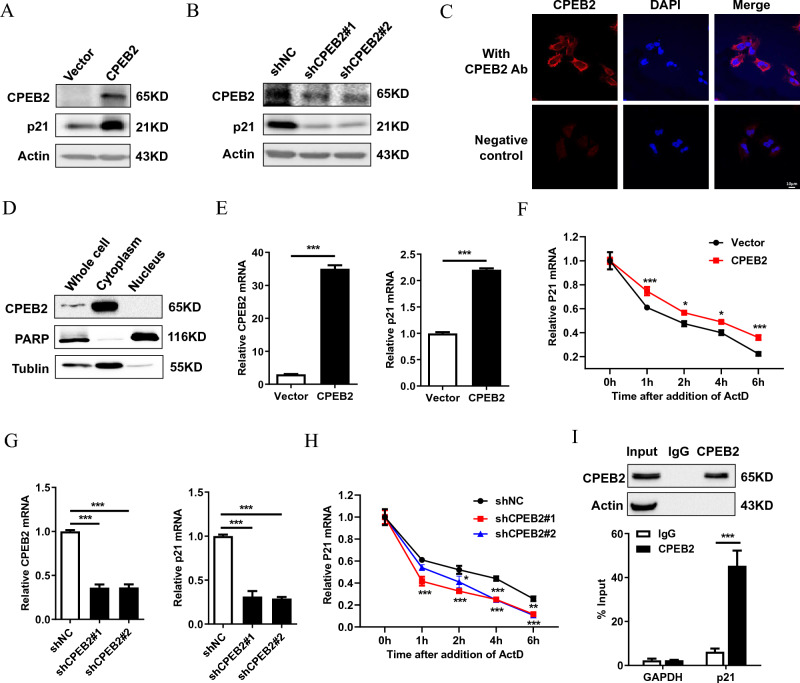
CPEB2 upregulates p21 expression by increasing its mRNA stability. (**A**,**B**) The protein expression of p21 in CPEB2 overexpression or knockdown U87 cells were detected by western blot. (**C**) The localization of CPEB2 in CPEB2 overexpression U87 cells was detected by indirect immunofluorescence assay. (**D**) The nuclei and cytoplasm of U87 cells were isolated, and CPEB2 protein expression levels were detected by western blot. (**E**,**G**) RT-qPCR of p21 mRNA expression in CPEB2 overexpression or knockdown U87 cells. (**F**,**H**) The levels of p21 transcript was measured by RT-qPCR in CPEB2 overexpression or knockdown U87 cells treated with 10 μg/mL actinomycin D for the indicated times. (**I**) CPEB2 interaction with p21 transcript in vitro. The lysates of CPEB2 overexpression U87 cells were immunoprecipitated with CPEB2 antibodies or control IgG, and RT-qPCR was used to measure the transcript levels of p21 and GAPDH precipitated by CPEB2 or IgG immunocomplexes. All values are mean ± SEM. Two way ANOVA followed by Tukey's multiple comparison test or Student t-tests were used. *P < 0.05, **P < 0.01, and ***P < 0.001. *SEM* standard error of the mean.

CPEBs regulate mRNA stability and translation by promoting cytoplasmic polyadenylation through binding the cytoplasmic polyadenylation element (CPE; with a consensus sequence of UUUUUAU) in the 3′-untranslated regions (3′-UTR) of target mRNAs to^32^. Interestingly, the 3 '-UTR of p21 contains a CPE signal indicating that p21 is a potential target of CPEBs. CPEB2 overexpression obviously increased p21 protein expression, while CPEB2 knockdown decreased p21 protein expression (Fig. 3A,B). Then, we investigated whether CPEB2 regulates p21 by targeting p21 mRNA. Given that mRNA is mainly degraded in the cytoplasm, we first detected the localization of CPEB2. Immunofluorescent staining results showed that CPEB2 is mostly localized in the cytoplasm (Fig. 3C), and nucleo-cytoplasmic separation experiments further confirmed this result (Fig. 3D). In line with this, CPEB2 overexpression resulted in an increase of p21 mRNA levels, while CPEB2 knockdown had an opposite effect (Fig. 3E,G). Next, we verified whether the changes in p21 transcription expression induced by CPEB2 are due to altered mRNA stability. To this end, we used actinomycin D to inhibit de novo mRNA synthesis. The results showed that CPEB2 overexpression stabilized p21 mRNA, while CPEB2 knockdown accelerated the degradation of p21 mRNA (Fig. 3F,H). In addition, RIP results showed that CPEB2 was correlated with p21 transcripts, but not with GAPDH transcripts (Fig. 3I). In conclusion, these findings indicate that CPEB2 could bind with p21 transcripts and upregulate p21 expression by increasing p21 mRNA stability.

### The inhibitory function of CPEB2 on glioma growth is partially dependent on upregulating p21 in vitro and in vivo

**Figure 4:**
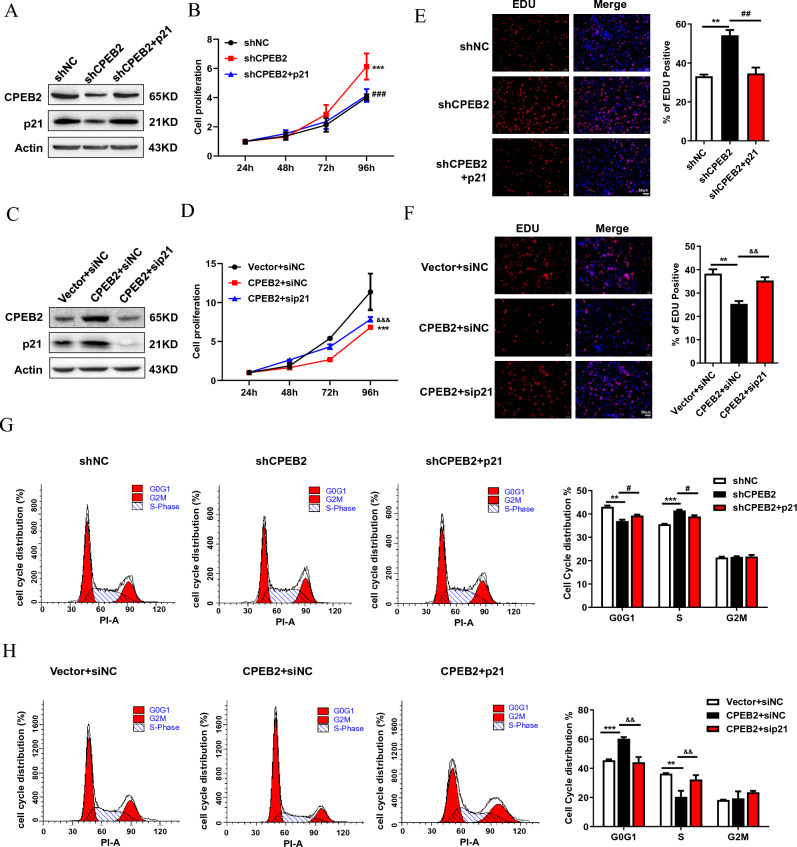
The anti-tumor effect of CPEB2 is partially dependent on upregulating p21 in glioma. (**A**) p21 overexpression plasmids were transfected into CPEB2 knockdown U87 cells. CPEB2, p21 and Actin protein expression were detected by western blot. (**B**) p21 overexpression abrogated the pro-proliferative effect of CPEB2 knockdown in CCK-8 assay. (**C**) p21 siRNA were transfected into CPEB2 overexpression U87 cells. CPEB2, p21 and Actin protein expression were detected by western blot. (**D**) silencing of p21 reversed glioma cell proliferation inhibition of CPEB2 overexpression in CCK-8 assay. (**E**,**F**) Indicated cells were stained with EDU and DAPI. The red color indicates EDU-positive nuclei. Column graph was mean ± SEM of three independent experiments. (**G**) p21 overexpression abrogated the G1/S phase transition effect of CPEB2 knockdown in flow cytometry assay. (**H**) silencing of p21 reversed the G1 cell cycle arrest effect of CPEB2 overexpression in flow cytometry assay. All values are mean ± SEM. Two way ANOVA followed by Tukey's multiple comparison test or Student t-tests were used. **P < 0.01, ***P < 0.001 *vs* vector or shNC; ^#^*P* < 0.05, ^##^*P* < 0.01, ^###^*P* < 0.001 *vs* shCPEB2; ^&&^*P* < 0.01, ^&&&^*P* < 0.001 versus CPEB2 + siNC. *SEM* standard error of the mean.

To validate the role of p21 in CPEB2-mediated anti-tumor effect, we overexpressed p21 in CPEB2-knockdown cells (Fig. 4A). As we expected, the data revealed that forced expression of p21 eliminated the pro-proliferative effect of CPEB2 knockdown by CCK-8 assays (Fig. 4B), Edu assays (Fig. 4C). We further depleted p21 expression in CPEB2-overexpressing cells (Fig. 4D). In accordance with previous results, p21 knockdown reversed the growth inhibition effect (Fig. 4E,F) caused by CPEB2-overexpression. Furthermore, the results of cell cycle analysis also verified the involvement of p21 in the CPEB2-mediated G1 cell cycle arrest (Fig. 4G,H).

**Figure 5:**
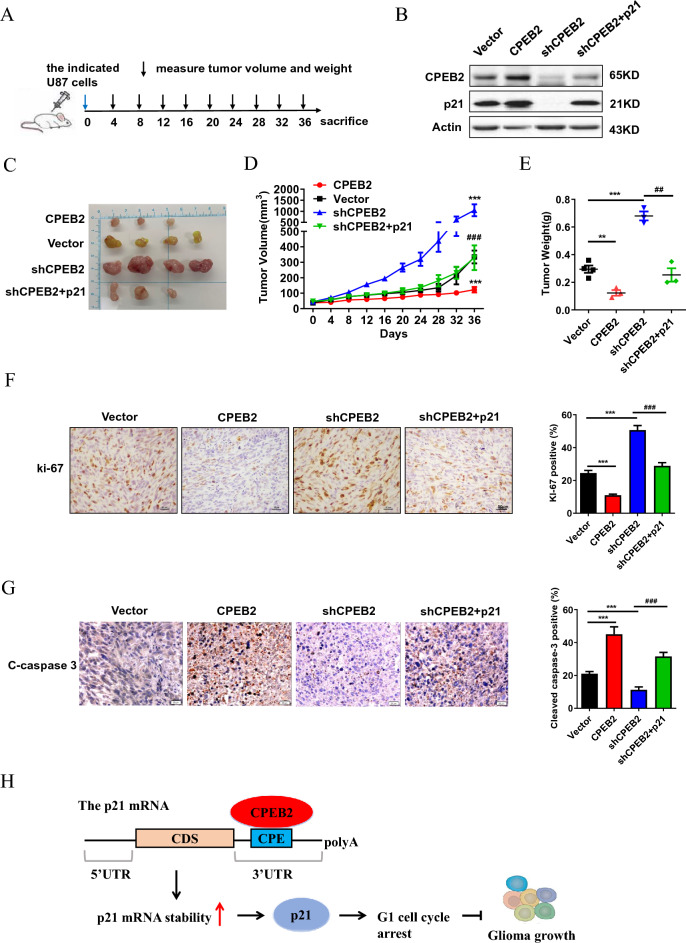
CPEB2 inhibits glioma growth partially through upregulating p21 in vivo. (**A**) Scheme of the experimental design. (**B**) The indicated stable expressing cells used for the in vivo study were confirmed by western blot analysis. (**C**) Images of the tumors from the indicated group. (**D**) Time course of tumor growth in mice. The indicated stable expressing cells were injected into nude mice, and tumor volumes were measured every 4 days. (**E**) Tumor weights were measured after the tumors were surgically dissected. (**F**,**G**) Ki-67 and cleaved-caspase 3 (C-caspaase 3) expression of the indicated tumor sections were detected by immunochemistry staining. Representative images were shown (×400 magnification), and ki-67 positive cellular index was defined as the presence of nuclear staining. (**H**) A schematic diagram of the function of CPEB2 in glioma. All values are mean ± SEM. Two way ANOVA followed by Tukey's multiple comparison test or Student t-tests were used. **P < 0.01, ***P < 0.001 versus vector and ^###^*P* < 0.001 versus shCPEB2. *SEM* standard error of the mean.

To determine the function of CPEB2 and the involvement of p21 in vivo, we injected the indicated stable U87 cells into BALB/c nude mice subcutaneously as shown in Fig. 5A, and the expression level of CPEB2 and p21 protein were confirmed by WB analysis (Fig. 5B). In vivo experiments showed that the tumor size, tumor growth rate, and tumor weight were significantly lower in the CPEB2 overexpression group than in the control group and that CPEB2 knockdown considerably promoted tumor growth, while the forced expression of p21 could abrogate tumor growth promotion caused by CPEB2 knockdown in vivo (Fig. 5C–E). We finally confirmed CPEB2 inhibited tumor proliferation and enhanced apoptosis by performing IHC staining for ki67 and cleaved-caspase 3 (Fig. 5F,G). Taken together, these results demonstrate that CPEB2 induces G1 cell cycle arrest and inhibits glioma cell proliferation by upregulation of p21.

## Discussion

In order to explore the role of CPEB2 in the occurrence and development of glioma, we first analyzed the expression levels of CPEB2 in glioma patient cohorts, and identified that CPEB2 was significantly downregulated in glioma. We further studied its role in glioma cell proliferation by CPEB2 overexpression or knockdown, and we demonstrated that CPEB2 could inhibit glioma cells proliferation in vivo and in vitro. Mechanistically, CPEB2 upregulates p21 expression by increasing its mRNA stability, thus further inducing cell cycle arrest. We conclude that CPEB2 is a novel tumor surpressor gene, inhibiting cancer hallmarks of proliferation while promoting apoptosis in glioma cells (Fig. 5H). Taken together, our findings indicate that CPEB2 could be served as a new anticancer therapeutic target in glioma treatment.

The function of CPEB2 in cancer appears to be complex and paradoxical, depending on the expression of different CPEB2 isoforms. UniProt database showed that CPEB2 has 8 isoforms (from Q7Z5Q1-2 to Q7Z5Q1-9) produced by alternative splicing, and the isoform Q7Z5Q1-2 has been chosen as the canonical sequence. In this study, we first found decreased levels of CPEB2 in glioma, and then we proved that CPEB2 could inhibit glioma cells proliferation through upregulating p21 by specifically overexpressing the canonical isoform Q7Z5Q1-2. Therefore, we only focused on the function and mechanism of the canonical isoform Q7Z5Q1-2 in this study, and whether different CPEB2 isoforms have contradictory function in glioma remains to be further investigated in the future.

CPEB family proteins bind the 3′-UTR of mRNAs that contain CPE elements^11,33^ and regulate their translation into proteins. The RRMs of CPEB2–4 share 97% sequence identity between them, and they appear to recognize the same cis-acting elements to regulate overlapping populations of mRNAs^12,13,15^. We previously reported that CPEB4 overexpression significantly upregulated tumor suppressor p21 and induced G1 cell cycle arrest in RCC^21^. Our data in this study showed that CPEB2 also induces G1 cell cycle arrest and inhibits cell proliferation in glioma. We hypothesized that CPEB2 modulates the cell cycle by regulating p21 in glioma. p21 was found to be overexpressed in the majority of gliomas while expressed at very low levels in normal glial cells^34^. Firstly, we screened cell cycle key factors in cells overexpressing CPEB2, and found that p21 mRNA was significantly upregulated by CPEB2 overexpression. Finally, we proved that CPEB2 upregulated p21 expression through increasing p21 mRNA stability, and the proliferation suppression ability of CPEB2 is partially dependent on upregulating p21 and cell cycle progression in glioma. Furthermore, different CPEBs may play diverse roles in cancer although CPEB2-4 are closely related^9^, and several studies have reported the expression and correlation of CPEB3 and CPEB4 in glioma^35,36^. Therefore, the synergistic and antagonistic effects of different CPEB family members in glioma needs to be further investigated in the future.

We further examined whether CPEB2 affect glioma cell apoptosis. Flow cytometry analysis showed that CPEB2 could promote glioma cell apoptosis in U87 cells pretreated with Cisplatin. Our results showed that CPEB2 inhibited cell proliferation by upregulation of p21, but the regulation of apoptosis by p21 has been demonstrated in a paradoxical manner^37–39^, so the specific mechanism of promoting glioma cell apoptosis by CPEB2 needs further discussion.

## Conclusions

In summary, we confirmed that CPEB2 is down-regulated in various glioma patient cohorts. Moreover, CPEB2 inhibits glioma cell proliferation partially dependent on upregulating p21 and further inducing G1 cell cycle arrest. This study highlights a mechanism underlying which CPEB2 inhibits giloma cell growth and provides a potential therapeutic target in patients with glioma.

## Methods

### Cell culture and reagents

Human glioma cell lines U87, U251 and HEK-293T was obtained from the Shanghai Cell Bank of the Chinese Academy of Sciences, which were cultured in Dulbecco’s modified Eagle’s medium (DMEM) with 10% (v/v) fetal bovine serum (FBS). And the cells were grown in a humidifed atmosphere containing 5% CO_2_ at 37 °C. The drugs used in this research were as follows: actinomycin D (HY-17559, MedChemExpress, USA) and Cisplatin (S1166, Selleck, USA).

### Glioma patient data analysis

The giloma patients data were analyzed using several public datasets such as GlioVis (http://gliovis.bioinfo.cnio.es), UALCAN (https://ualcan.path.uab.edu/index.html), Gene Expression database of Normal and Tumor tissues 2 (GENT2) database (http://gent2.appex.kr/gent2/) and cBioPortal database (https://www.cbioportal.org/).

### Transfection

To inhibit CPEB2 expression with siRNA, 30 pmol of CPEB2-targeting siRNA or nonspecific control (NC) were transfected into the 6-well cells with 30–50% confluence for 48 h, using 2.5 µl siLentFect™ lipid reagent (Bio-Rad, 1703361) according to the manufacturer's instructions. The sequences of CPEB2-targeting siRNA or NC were as follows: si-CPEB2#1 (GCGAGUUGCUUUCUCCAAUTT); si-CPEB2#2 (GGAACUAUGAAUCAGAUAUTT) and si-NC (UUUUCCGAACGUGUCACGUTT). CPEB2 was subcloned into lentiviral vector pCD513B, and then packaged virus in HEK-293T cells to infect U87 and U251 cells. Cells were separately transfected with the two CPEB2 shRNAs to stabilize the knockdown of CPEB2, and then selected in the presence of puromycin. The target sequences were as follows: sh-CPEB2#1 (GTGTTCAGAACAGACAACAAT) and sh-CPEB#2 (GAGTTCCATAAGCCATTGGTA).

### WB analysis

0.5% NP-40 buffer was added to the cells and then lysed for 30 min until the cells were completely ruptured. The supernatant proteins were carefully aspirated after centrifugation at 13,000 rpm for 10 min. SDS-PAGE gel electrophoresis were used to separate the equal amounts (40 μg) of proteins. The blots were cut prior to hybridisation with specific antibodies according to the protein molecular weight during blotting. The immune complex on the membrane was detected with the ECL kit (Termo Scientifc, 35,055) after incubation with the specific antibody. The used antibodies were acquired from commercial sources: rabbit polyclonal anti-CPEB2 (NBP2-56392, Novus Biologicals, USA, 1:1000), and rabbit monoclonal anti-p21 (2947s, Cell Signaling, USA, 1:1000).

### RNA extraction and qRT-PCR

1 ml TRIzol reagent (15596026, Invitrogen, USA) were used to extract total RNA from the p-100 cultured cells and tissues according to the manufacturer's instructions. PrimeScript reverse transcription (RT) reagent kit (R323, Vazyme, China) was used to synthesize complementary DNA in the presence of gDNA Eraser. The Q-PCR reaction was performed according to the instructions of SYBR Green Master Mix Kit (Q331-02, Vazyme, China), and results were calculated according to the Ct value (2^ΔΔCt^). All the primers were listed as follows: CPEB2 forward 5′-GGTGGTCTTCCTCCAGATAT-3′ and reverse 5′-GAGGCCAATCTACTACCAAA-3′; GAPDH 5′-forward GGTGGTCTCCTCTGACTTCAACA-3′ and GAPDH 5′-reverse GTTGCTGTAGCCAAATTCGTTGT-3′; p21 forward 5′-GTCAGAACCCATGCGGCAGCAAG-3′ and reverse 5′-CAGGTCCACATGGTCTTCCTCTG-3′.

### Immunofluorescence staining

4% paraformaldehyde (P0099, Beyotime) was used to fix the CPEB2 overexpression U87 cells grown on coverslips in a 24-well plate for 25 min, and then the cells were treated with 0.1% Triton X-100 solution on ice for 5 min. After that, cells were sealed with 3% BSA for 1 h followed by incubation with the primary antibody against Flag (F1804, Sigma, USA, 1:500) at 4 °C overnight, and then the cells were incubated with fluorescently labeled secondary antibodies for 1 h after washing with phosphate-buffered saline (PBS) for 5–10 min. The nuclei was stained by DAPI (4’,6-diamidino-2-phenylindole) for 5 min. Zeiss Axio observer confocal microscope was used to capture images after the slides are mounted with 90% glycerin.

### Immunohistochemistry staining (IHC)

The xenograft tumor tissues which were paraffin-embedded were punched to 1.5 mm diameter cores. According to the streptavidin-peroxidase method using a standard Sp Kit (PV-9001, Zhongshan biotech, Beijing, China). The standard protocol for immunohistochemistry staining was described previously in our lab^40^. Rabbit polyclonal Ki-67 antibody (12202 s, Cell Signaling, USA, 1:500) and rabbit monoclonal cleaved Caspase-3 antibody (9664 s, Cell Signaling, USA, 1:2000) were used as primary antibody and incubated overnight at 4 °C. We used PBS instead of primary antibody as negative control. All images were recorded by Olympus BX-51 light microscope. Ki-67 positive cell index was described as the presence of nuclear staining. All stained nuclei were rated positive regardless of staining intensity. Cell counts were performed in 5 randomly selected fields using a conventional light microscope. The calculation formula is as follows^41^.

### Cell proliferation and colony formation assays

Cell counting Kit 8 (CCK-8) (C0038, Beyotime, China) was used to perform the cell proliferation assay. 200 cells were seeded into 6-well plates for the colony formation assay. The plates were washed with PBS for 3 times after 14 days of incubation, and then stained with 0.5% crystal violet for 20 min after fixed with 70% ice methanol for 20 min. ImageJ software was used to count the number of colonies. The experiment was repeated three times independently.

### 5-Ethynyl-20-deoxyuridine (EdU) assay

10,000 cells were grown overnight on 96-well plates. 50 µM EdU (C10310, RiboBio, China) was added to each well and incubated cells (5% CO_2_, 37 °C) for 2 h. The plates were washed with PBS twice, and then treated with 0.5% TritonX-100 for 20 min after fixing with 4% paraformaldehyde for 30 min. Next, cells were stained with 100µL Apollo dye solution for 30 min. Finally, the nuclei were stained with DAPI for 30 min. The proportion of cells bound to EdU was determined by fluorescence microscopy.

### Xenograft assay

The animal studies were approved by the laboratory animal ethics committee of Xuzhou Medical University in accordance with institutional and Chinese government guidelines for animal experiments. To evaluate the effect of CPEB2 on tumorigenesis in vivo, the indicated U87 cells (1 × 10^7^ cells in 100 µL serum-free medium containing 0.25 v/v Matrigel) were subcutaneously injected into 6-week-old female BALB/C nude mice (Beijing HFK Bioscience, China). Tumor volume was measured every 3–4 days, and was calculated according to the following formula: *V*(mm^3^) = length × width^2^ × 0.5. The tumors were carefully removed, imaged, and weighed at the end of the experiment when the mice were sacrificed by carbon dioxide euthanasia.

### Cell cycle and cell apoptosis assay

The cells were collected and fixed with 70% ice ethanol overnight. The fixed cells were incubated with PBS containing 400 μg/ml propidium iodide and 20 mg/mL RNaseA at room temperature for 30 min after being washed three times with PBS. The FACSCanto flow cytometer (BD Biosciences, San Jose, CA) and the ModFit LT 3.0 software were used to analyze the samples. For cell apoptosis assay, 5 × 10^5^ cells were collected and incubated in 500 mL of binding buffer after treated with 15 mg/ml Cisplatin for 24 h, and then the cells were added 5 μL Annexin V-EGFP and 5 μL Propidium Iodide in turn, and mixed well for 30 min at room temperature in the dark. Finally, the apoptosis of these cells was measured by flow cytometry.

### RNA immunoprecipitation (RIP)

Lysis buffer (containing 5 mM PIPES. pH 8.0, 85 mM KCl, 0.5% NP40, 1% SDS, 10 mM EDTA, and 50 mM Tris–HCl, pH 8.1) supplemented with a protease inhibitor cocktail and an RNase inhibitor (N8080119, Thermo Fisher Scientific, USA) was used to harvest cells. After pre-washed with protein A/G agarose beads (P2055, Beyotime, China), cell lysates were then incubated overnight at 4 °C with indicated antibodies or IgG control on a rotator and. The antibody-RNA complexes were collected and then the immunoprecipitated RNA was extracted for qRT-PCR analysis.

### Statistical analysis

GraphPad Prism software (version 9.0) was used to calculate the statistical results of mean ± SEM in the experiment. Two-way ANOVA followed by Tukey post-hoc test for multiple comparisons or two-tailed unpaired student t-test were performed to analyze datasets. A probability (p) value of < 0.05 was considered statistically significant.

### Ethics approval

All animal studies were approved by the Xuzhou Medical University’s laboratory animal ethics committee and Chinese government guidelines for animal experiments. All methods used in animal experiments were carried out in accordance with relevant guidelines, and handled according to standard use protocols and animal welfare regulations, and we also confirmed that all animal methods were reported in accordance with ARRIVE guidelines (http://arriveguidelines.org) for the reporting of animal experiments.

### Supplementary Information

Supplementary Information.

## Data Availability

The data and materials presented in this study are available on request from the corresponding author.

## Abbreviations

CPEB2: Cytoplasmic polyadenylate element-binding protein 2
CNS: Central nervous system
CPEBs: CPEB family proteins
RRMs: RNA recognition motifs
ZF: Zinc finger
TNBC: Triple-negative breast cancer
TMZ: Temozolomide
FBS: Fetal bovine serum
WB: Westernblot
RT: Reverse transcription
qRT-PCR: Reverse transcription‑quantitative polymerase chain reaction
PBS: Phosphate-buffered saline
DAPI: 4′,6-Diamidino-2-phenylindole
IHC: Immunohistochemistry
CCK-8: Cell counting Kit 8
EdU: 5-Ethynyl-20-deoxyuridine
RIP: RNA immunoprecipitation
GENT: Gene Expression database of Normal and Tumor tissues
CPE: Cytoplasmic polyadenylation element
3′-UTR: 3′-Untranslated regions
