# Supplementary material for: CPEB2 inhibit cell proliferation through upregulating p21 mRNA stability in glioma
Source: Sci Rep. 2023 Dec 29;13:23103. doi: 10.1038/s41598-023-50848-0 (PMC10756880; doi:10.1038/s41598-023-50848-0)
Supplement: Supplementary file 1 — Supplementary Information. [file 41598_2023_50848_MOESM1_ESM.docx]

**
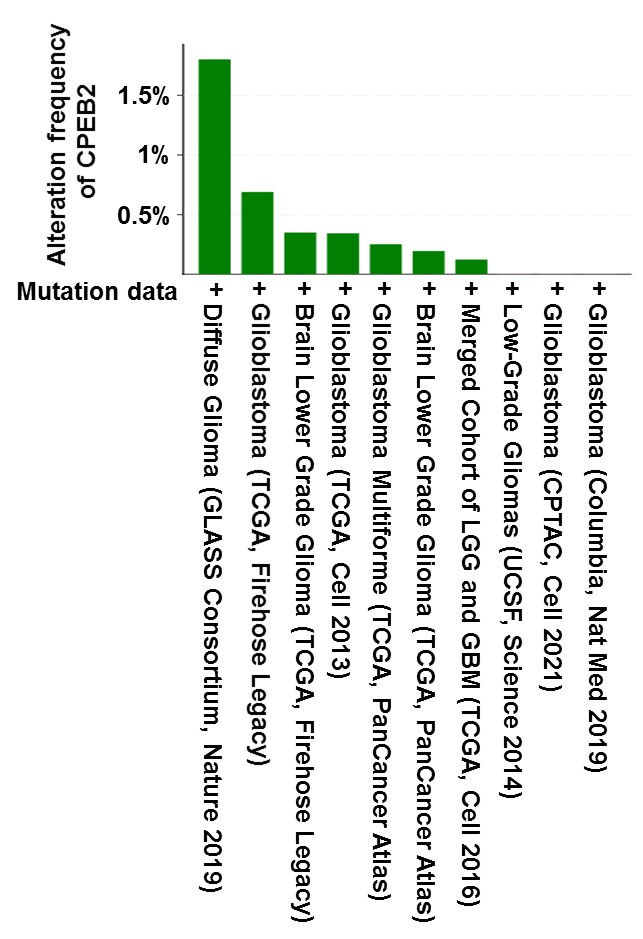
**

**Fig S1. The mutation rate of CPEB2 in glioma patients are very low in cBioPortal database.**

**
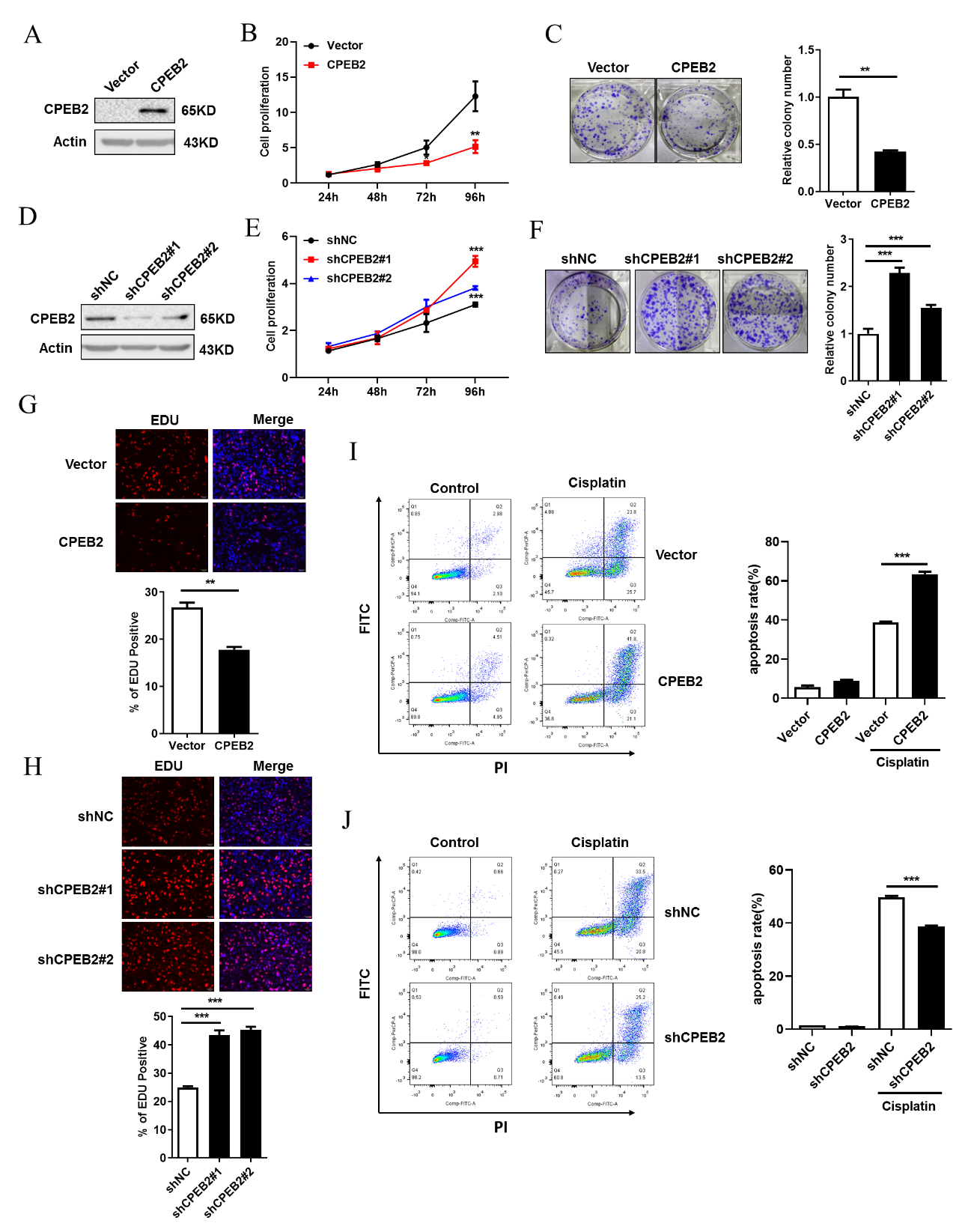
**

**Fig S2. CPEB2 inhibits cell proliferation and promotes apoptosis in U251 cells.**

**A** The efficiency of CPEB2 overexpression in U251 cells. **B** Cell growth of U251 cells with CPEB2 overexpression or not was detected by CCK-8 assays after 1, 2, 3 and 4 days (n=5). **C** Colony formation of U251 cells with CPEB2 overexpression or not. **D** The efficiency of CPEB2 knockdown in U251 cells. **E** Cell growth of U251 cells with CPEB2 knockdown or not was detected by CCK-8 assays after 1, 2, 3 and 4 days (n=5). **F** Colony formation of U251 cells with CPEB2 knockdown or not. **G** and **H** Indicated U251 cells were stained with EDU and DAPI. The red color indicates EDU-positive nuclei. The statistical analysis of EDU staining was performed by Image-Pro Plus 6.0 software (n =3). Column graph was mean ± SEM of three independent experiments. **I** and **J** Flow cytometry detection of apoptosis with Annexin V/PI staining. U251 cells with CPEB2 overexpression or knockdown were treated with 25 μg/ml cisplatin for 24 hrs. Apoptotic cells were detected and quantified with flow cytometry. All values are mean ± SEM. Two way ANOVA followed by Tukey's multiple comparison test or Student t-tests were used**P* < 0.05, ***P* < 0.01, and ****P* < 0.001. SEM, standard error of the mean.


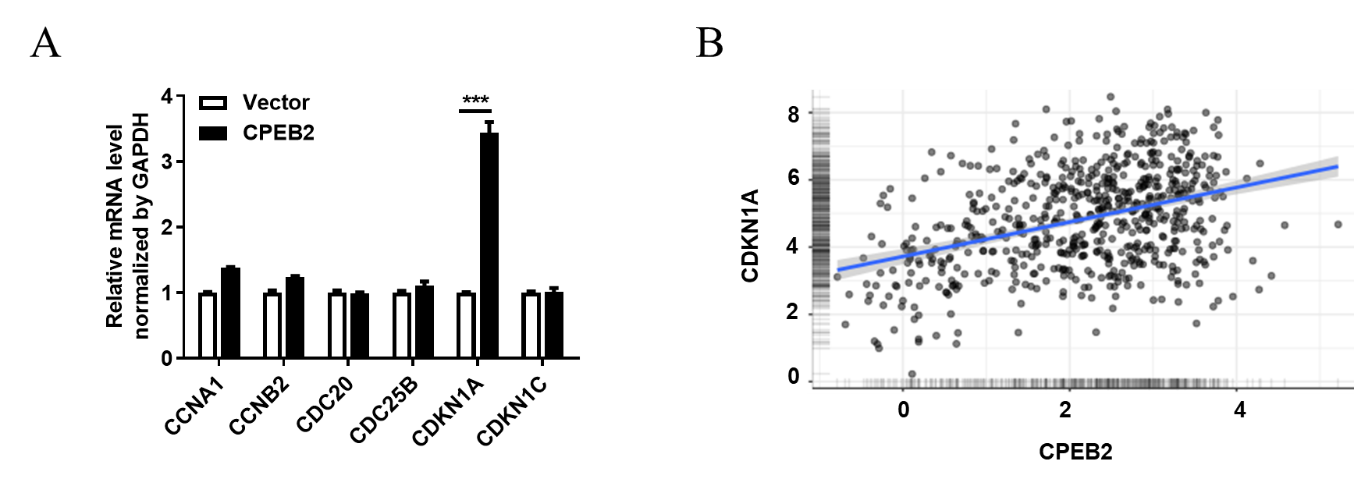


**Fig S3. CPEB2 upregulates and positively correlated with CDKN1A (p21) mRNA.**

**A** Cell cycle-related genes including CDKN1A (p21) were detected by RT-qPCR in U87 cells. **B** CDKN1A (p21) expression is positively associated with CPEB2 expression in glioma from CGGA database (*P* =0.00, r =0.39). All values are mean ± SEM. Student t-tests were used. ****P* < 0.001. SEM, standard error of the mean.

**Fig S4. The original images of blots.**

**Figure 1H**

**CPEB2**

**Vector**


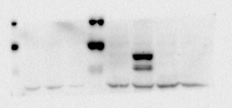

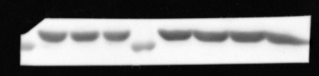

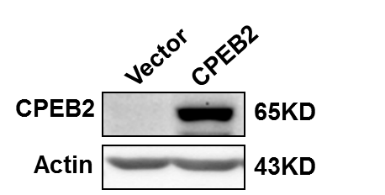


**40kd**

**55kd**

**70kd**

**Actin**

**CPEB2**

**Figure 1I**

**shNC**

**shCPEB2#1**

**shCPEB2#2**


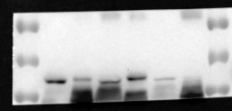

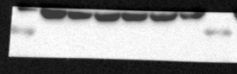

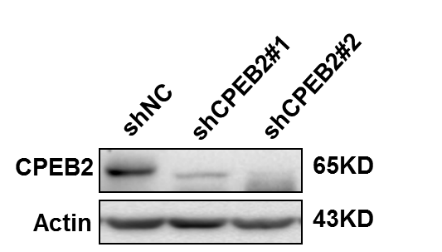


**70kd**

**55kd**

**40kd**

**Actin**

**CPEB2**

**Figure 1P**

**
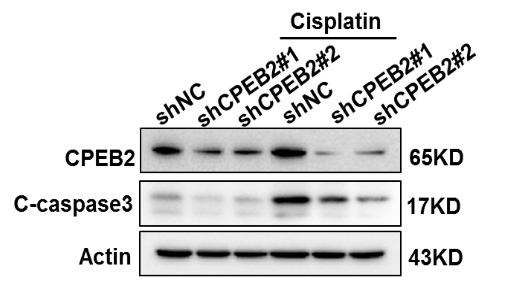

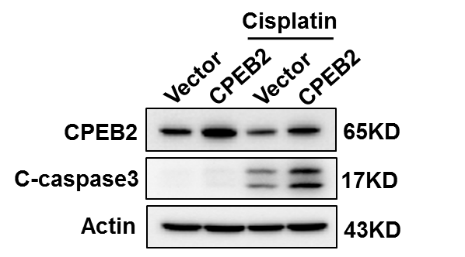

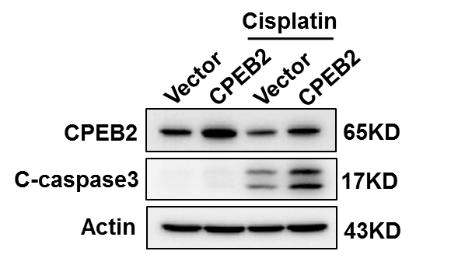
**


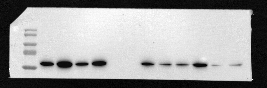


**55kd**

**70kd**


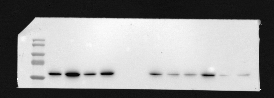


**Figure 1Q**

**
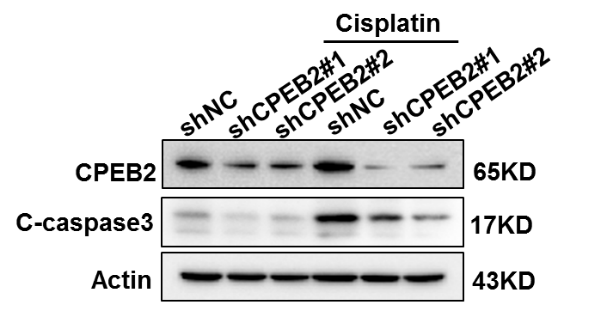
**


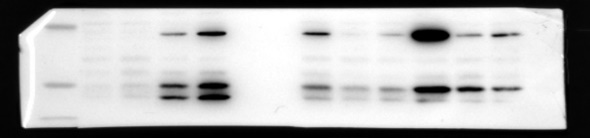


**15kd**


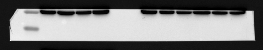


**40kd**

**Figure 3A**

**Vector**

**CPEB2**


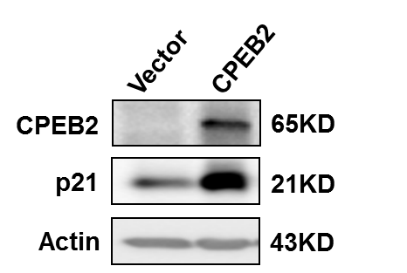


**p21**

**55kd**

**70kd**

**25kd**

**CPEB2**


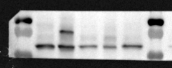

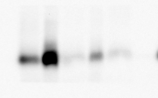

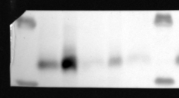

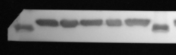


**15kd**

**Actin**

**40kd**

**Figure 3B**

**shCPEB2#2**

**shCPEB2#1**

**shNC**


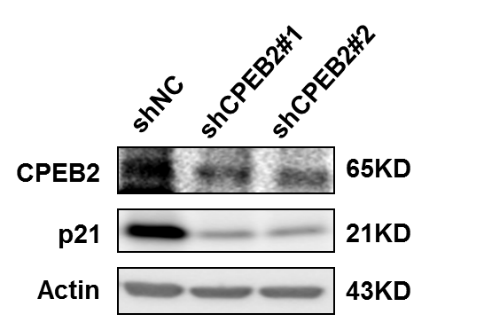

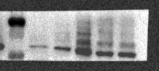

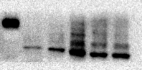


**70kd**

**CPEB2**

**55kd**


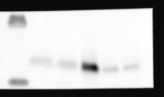


**Actin**

**40kd**

**15kd**

**25kd**

**p21**


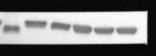


**Figure 3D**


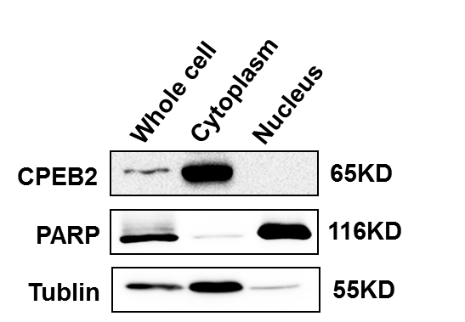


**Tubulin**

**70kd**

**55kd**

**55kd**

**130kd**

**100kd**

**PARP**

**CPEB2**


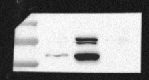


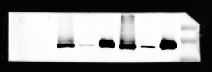


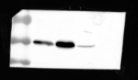


**Figure 3I**


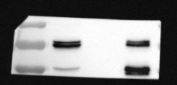


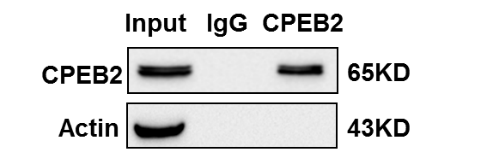


**CPEB2**

**70kd**

**55kd**

**40kd**

**Actin**


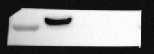


**Figure 4A**


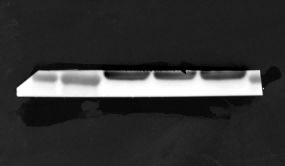

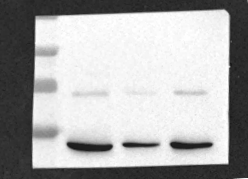

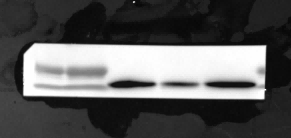

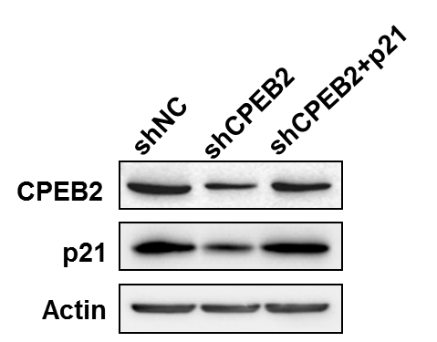


**Actin**

**CPEB2**

**70kd**

**p21**

**Figure 4C**


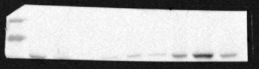


**Vector+siNC**

**CPEB2+sip21**

**CPEB2+siNC**

**Actin**

**40kd**

**75kd**

**15kd**

**25kd**

**p21**

**CPEB2**


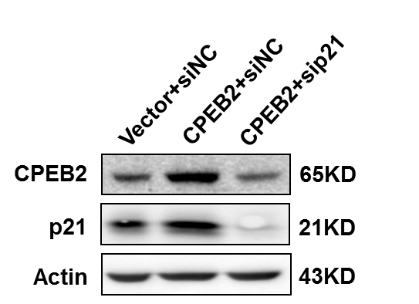


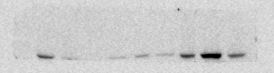


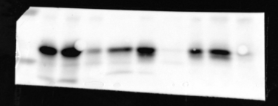


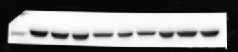


**Figure 5B**

**shCPEB2+p21**

**CPEB2**

**shCPEB2**

**Actin**

**40kd**

**70kd**

**55kd**

**25kd**

**15kd**

**p21**

**CPEB2**


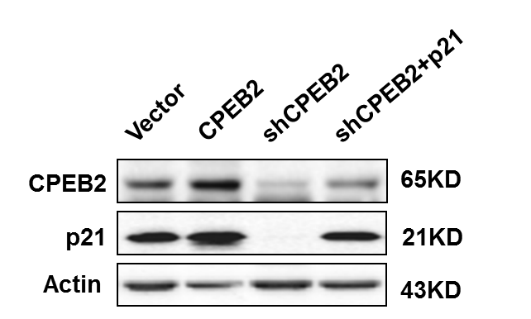


**Vector**


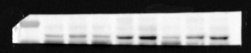


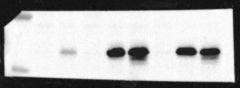


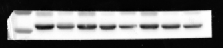


**Fig S2A**

**CPEB2**

**Vector**


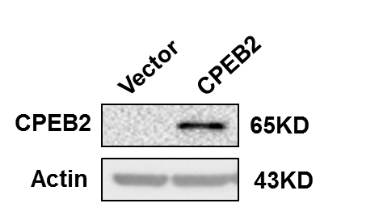

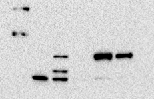

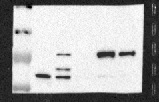


**70kd**

**CPEB2**

**40kd**

**55kd**

**Actin**


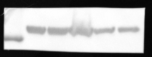


**Fig S2D**


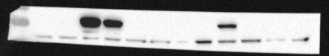

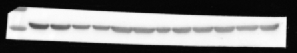


**shCPEB2#1**

**shCPEB2#2**

**shNC**


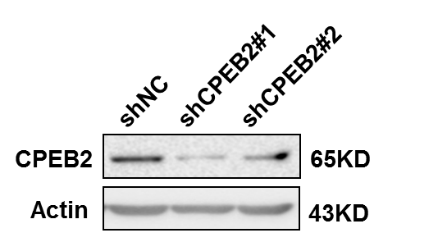


**40kd**

**70kd**

**CPEB2**

**Actin**
